# The Situation of Counterfeited and Mislabeled Commercialized Edible Mushrooms in China and the Development of Possible Controls

**DOI:** 10.3390/foods13193097

**Published:** 2024-09-27

**Authors:** Jinlin Liu, Jingyi Sun, Ruyan He, Jing Xia, Peimin He

**Affiliations:** 1 State Key Laboratory of Marine Geology, Tongji University, Shanghai 200092, China; jlliu@tongji.edu.cn; 2 College of Oceanography and Ecological Science, Shanghai Ocean University, Shanghai 201306, China; sjyyjs2009@163.com (J.S.); hryhry9@163.com (R.H.); 3 School of Oceanography, Shanghai Jiao Tong University, Shanghai 200030, China

**Keywords:** edible fungi, yield, edible and medicinal value, counterfeit and low-quality products, poisonous wild mushrooms, DNA barcoding

## Abstract

Edible mushroom products, encompassing both cultivated and wild varieties, are highly favored by consumers due to their rich nutritional profiles, including significant levels of proteins and amino acids. These mushrooms have extensive applications across the food, pharmaceutical, and cosmetic industries, making the edible mushroom industry a vital component of global poverty alleviation efforts. Taking China as an example, the country produces over 45 million tons of edible mushrooms annually, accounting for 94.01% of the world’s total production, thereby establishing itself as the leading global producer of edible mushrooms. However, alongside the rapid expansion of this industry, concerns have emerged regarding counterfeit products and incidents of poisoning resulting from the consumption of toxic wild mushrooms. As follows, to advance the development and integrity of the mushroom production and processing industry: (1) This study presents the situation of counterfeit edible mushrooms and elucidates the factors contributing to the production of fraudulent products from both subjective and non-subjective perspectives. (2) We provide a detailed introduction to 22 varieties of freshly cultivated edible mushrooms and commonly encountered wild edible mushrooms in the Chinese consumer market, proposing the application of DNA barcoding, environmental DNA analysis, and other technologies for the future authentication of counterfeit mushroom products. (3) Concurrently, we present an overview of mushroom poisoning incidents in China from 2010 to 2023, emphasizing the challenges in mitigating the risks associated with wild mushroom consumption and preventing food poisoning, thereby necessitating heightened consumer caution. (4) Finally, we offer four recommendations aimed at ensuring the healthy, stable, and sustainable growth of the edible mushroom industry.

## 1. Introduction

The cultivation of edible fungi, rich in proteins and amino acids [1], has been vigorously promoted by the United Nations Development Program over the past three decades. In 2017, it was designated as a “China–UN Peace and Development Fund” project by the United Nations due to the anti-cancer, anti-aging, and other beneficial effects of these fungi [2]. According to statistical data from FAOSTAT [3], over 60 countries and regions worldwide have engaged in the commercial cultivation of edible fungi (Table S1). In 2022, the global market size for edible fungi exceeded USD 55 billion. China, as the largest producer of edible fungi [4], produces more than 45 million tons annually, accounting for 94.01% of the total global output (Table S1) and providing a significant quantity of edible fungus products and technological advancements for the global market [3].

Currently, with the support of the United Nations cooperation program, technicians from over 100 countries and regions are both being trained on and are actively promoting China’s ecological cultivation technology for edible fungi. For instance, the “mushroom-grass (Juncao) technology”, developed by Professor Zhanzhi Lin, not only facilitates mushroom cultivation, but also contributes to mitigating land desertification and improving saline–alkali soil conditions [5,6]. This technology has been globally promoted by the Chinese government for nearly 30 years and has achieved significant success. China is dedicated to addressing the food crisis in certain low- and middle-income nations [7] and advancing sustainable development in impoverished countries by promoting the cultivation of edible fungi through free foreign technical assistance. Countries such as South Africa, Rwanda, Kenya, Nigeria, Tanzania, and Lesotho have enhanced their food supply by adopting or optimizing their cultivation techniques for *Pleurotus ostreatus*, *Agaricus bisporus*, and *Lentinula edodes*.

**Figure 1:**
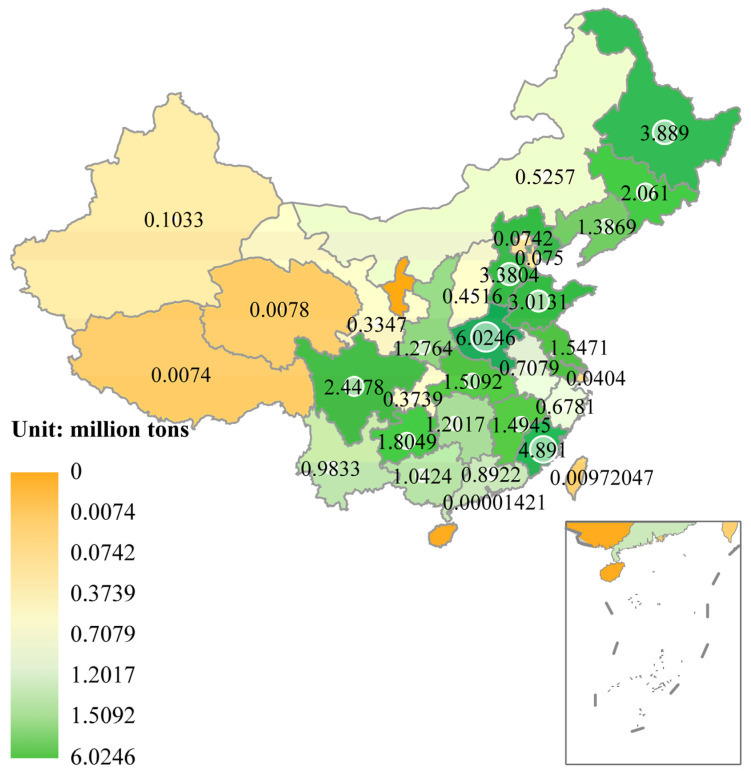
Production of edible mushrooms in various provinces, autonomous regions, municipalities, and special administrative regions of China in 2022.

Meanwhile, the domestic edible fungus planting industry in China has experienced rapid development, contributing positively to the promotion of rural revitalization [8]. More than 400 counties, accounting for 72% of the total number of national poverty-stricken counties in China, have opted to develop the edible fungus industry [9]. As the world’s largest producer of edible fungus (Table S1), there are numerous edible fungus-planting bases in central, southwest, southeast, and northeast China with substantial output. For example, Henan Province produces 602.46 million tons, Fujian Province produces 489.1 million tons, Heilongjiang Province produces 388.9 million tons, Hebei Province produces 338.04 million tons, Shandong Province produces 301.31 million tons, Sichuan Province produces 244.48 million tons, Jilin Province produces 206.10 million tons, Guizhou Province produces 180.49 million tons, and Jiangsu Province produces 154.71 million tons (Figure 1; Table S2). The primary cultivated varieties of edible fungi include *L. edodes*, *Auricularia auricula*, *P. ostreatus*, *Flammulina velutipes*, *Pleurotus eryngii*, and *A. bisporus*. These products are predominantly marketed within China. Chinese exports of edible fungi encompass mushroom mycelium, fresh and refrigerated fungi, salted fungi, dried fungi, and canned fungi. For instance, in 2022, the annual export volume of edible fungus products from China reached 682,500 tons. In the same year, the rest of the world (excluding China) produced approximately 2.90 million tons of edible fungus (Table S1). Chinese exports accounted for 19.06% of the global demand for edible fungus products outside China.

However, the burgeoning edible mushroom industry has encountered various challenges during its development. In addition to cultivated edible mushrooms, wild mushrooms are also favored by consumers; however, the accidental consumption of poisonous wild mushrooms has led to a series of poisoning incidents [10,11,12,13,14], resulting in significant fatalities. Furthermore, instances of counterfeit or low-quality products have emerged in mushroom markets [15,16,17,18,19,20,21,22,23], posing a genuine threat to the quality and safety of edible mushrooms and the sustainable development of the industry.

This study provides a systematic and comprehensive overview of the current status of counterfeit and low-quality edible mushrooms in China for the first time. It also analyzes both the subjective and non-subjective factors contributing to fraudulent sales. Additionally, scientific insights and solutions are proposed regarding market sales of edible mushrooms, identification methods for counterfeit and low-quality mushroom products, the importance of avoiding the consumption of poisonous wild mushrooms, and future development directions for the edible mushroom industry. This aims to provide references and suggestions for sustainable and healthy development within the mushroom industry.

## 2. Details of the Phenomenon of Selling Certain Fake Edible Mushrooms

### 2.1. Lentinula edodes, Wolfiporia cocos, and Polyporus umbellatus

**Figure 2:**
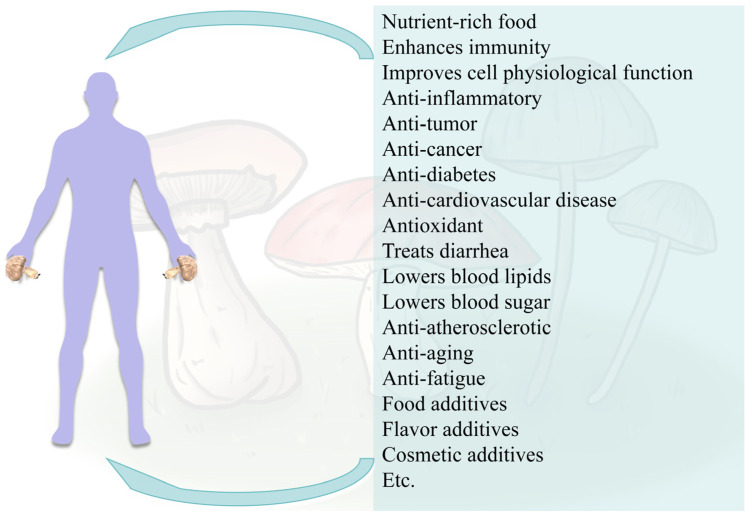
The main edible, medicinal, and industrial values of edible mushrooms.

*Lentinula edodes* pileus powder is a valuable commodity in the global agricultural market, renowned for its high nutritional content, including proteins and trace elements, as well as its medicinal properties such as anti-inflammatory, anti-cancer, and anti-diabetic effects (Figure 2). It is commonly utilized as an edible fungus, flavoring agent, or food supplement [22]. However, there have been instances of fraudulent practices in the market involving the substitution of low-quality *L. edodes* stipe powder, *P. eryngii* powder, or *P. ostreatus* powder for genuine *L. edodes* pileus powder (Table 1). *Wolfiporia cocos* is a traditional Chinese herbal medicine with demonstrated anti-tumor, anti-inflammatory, and immunomodulatory properties (Figure 2) [15]. While it is primarily used medicinally, it can also be consumed in small quantities. Counterfeit *W. cocos* products found on the market are sometimes produced by grinding *Oryza sativa* into rice flour and compressing the slices to mimic authentic *W. cocos* slices (Table 1). *Polyporus umbellatus* has been employed in traditional Chinese medicine for over two millennia [24]. Its edible fruiting body and medicinal underground sclerotium are frequently utilized to address conditions such as diarrhea and edema (Figure 2). Unfortunately, substandard *P. umbellatus* products have been observed in the market, where *P. umbellatus* is soaked in alum solution to artificially increase its weight (Table 1). Additionally, there are cases of *Agrocybe tuberosa* being used to imitate *P. umbellatus* [19].

### 2.2. Tricholoma matsutake, Ganoderma lucidum, Cantharellus cibarius, and Termitomyces albuminosus

*Tricholoma matsutake* is a highly prized edible fungus [18] that is popular in China, Japan, South Korea, and other countries. It contains valuable active compounds such as matsutake alcohol, matsutake polysaccharide, matsutake polypeptide, and isomatsutake alcohol, which exhibit anti-tumor, antiviral, anti-diabetic, anti-cardiovascular disease, antioxidant, and anti-aging properties and immune system activation effects (Figure 2). *Stropharia rugosoannulata*, *P. eryngii*, *Catathelasma ventricosum*, and *Agaricus blazei* are commonly used to counterfeit *T. matsutake* in the market (Table 1). *Ganoderma lucidum* spore powder possesses medicinal values, including anti-tumor activity and antioxidant properties [25], and it is widely utilized in food additives, drug development, and functional cosmetic additives (Figure 2). The inferior-quality *G. lucidum* spore powder found on the market often contains added dyed starch as an adulterant (Table 1), significantly diminishing its medicinal and nutritional value. *Cantharellus cibarius* and *Termitomyces albuminosus* have high edible and medicinal values with various effects such as reducing blood lipids, lowering blood sugar, and exhibiting anti-cancer properties (Figure 2) [26,27]; *Pleurotus citrinopileatus* and *Zizania latifolia* are often used in the market to fake *C. cibarius*; and *Oudemansiella raphanipies* is often used to fake *T. albuminosus* (Table 1).

### 2.3. Boletus purpureus and Ophiocordyceps sinensis

Despite the presence of neurotoxins in both *Boletus purpureus* and *Boletus luridus*, which can cause adverse effects such as vomiting and diarrhea in humans [28], the consumption of these locally abundant mushrooms remains popular in China due to their prized flavor and longstanding culinary tradition. Differential pricing in the market has resulted in instances of fraudulent substitution, with *B. luridus* (USD 8/kg) being passed off as *B. purpureus* (USD 35/kg) (Table 1). *Ophiocordyceps sinensis* (Dong Chong Xia Cao) is a highly valued traditional Chinese medicine, commanding a price higher than that of gold per gram due to its rarity and medicinal properties, particularly its active compounds, including cordycepin, mannitol, and ergosterol, which confer immune system regulation, anti-tumor, anti-fatigue, and anti-atherosclerotic functions (Figure 2) [29,30]. Currently, there are cases of using *Cordyceps militaris*, *Cordyceps hawkesii*, *Metacordyceps taii*, dough, and the root of the *Stachys geobombycis* to imitate *O. sinensis* in both domestic and foreign markets (Table 1), which has drawn the attention of market supervision and public security departments.

## 3. The Reasons for the Phenomenon of Selling Fake Edible Mushrooms

### 3.1. Subjective Factors in the Sale of Certain Fake Edible Mushrooms

Reason 1: Profit maximization. For instance, sellers often use inexpensive and lower-quality *A. blazei* and *S. rugosoannulata* to counterfeit *T. matsutake* (Table 1). It is challenging for consumers to visually distinguish between different dried edible fungus varieties, allowing sellers to manipulate consumer choices towards purchasing counterfeit products based on subjective criteria. Additionally, while the standard Chinese pinyin for *T. matsutake* is “Song Rong”, *A. blazei* and *S. rugosoannulata* are referred to as “Ji Song Rong” and “Chi Song Rong”, respectively. Sellers often deliberately sell fresh *A. blazei* and *S. rugosoannulata* as “Song Rong” to imitate authentic *T. matsutake* and seek profits.

As another example, *L. edodes* can be considered. *Lentinus edodes* has been cultivated in China for over 800 years and currently accounts for more than 90% of the global production of *L. edodes* [9]. Based on output data, there is no shortage of global consumer demand for *L. edodes*. However, some production enterprises engage in commercial fraud by mixing low-quality and low-cost *L. edodes* stipe powder, *P. eryngii* powder, or *P. ostreatus* powder with *L. edodes* pileus powder (Table 1), aiming to reduce production costs at the risk of legal repercussions.

### 3.2. Non-Subjective Factors in the Sale of Certain Fake Edible Mushrooms

Reason 2: Lack of clarity in standardized names. The lack of clarity among sellers regarding the standardized Chinese and scientific names of edible fungi contributes to the misidentification and mislabeling of products. For example, the standard Chinese pinyin for *O. raphanipies* is “Hei Pi Ji Zong”, often abbreviated as “Ji Zong” by sellers. However, *T. albuminosus* has the same abbreviation, “Ji Zong”, leading to *O. raphanipies* being mistakenly sold as *T. albuminosus*. Additionally, establishments such as restaurants and hotels commonly refer to dishes made with *O. raphanipies* as “Ji Zong”, further perpetuating the sale of mislabeled products resulting from non-subjective factors.

Reason 3: Inability to accurately identify species. The inability of pickers to accurately identify species of fungus can lead to the collection of poisonous wild mushrooms. Due to limited experience and identification skills, these poisonous wild mushrooms may be mistakenly identified as being edible. Subsequently, these poisonous wild mushrooms can enter catering service establishments (such as street stalls, staff canteens, restaurants, rural banquets, cafes, fast food stores, and school canteens) [11], resulting in a series of mushroom poisoning incidents.

## 4. Discussion and Conclusions

### 4.1. Further Standardization of the Nomenclature for Edible Fungi and the Establishment of a Comprehensive Identification System Are Essential

**Figure 3:**
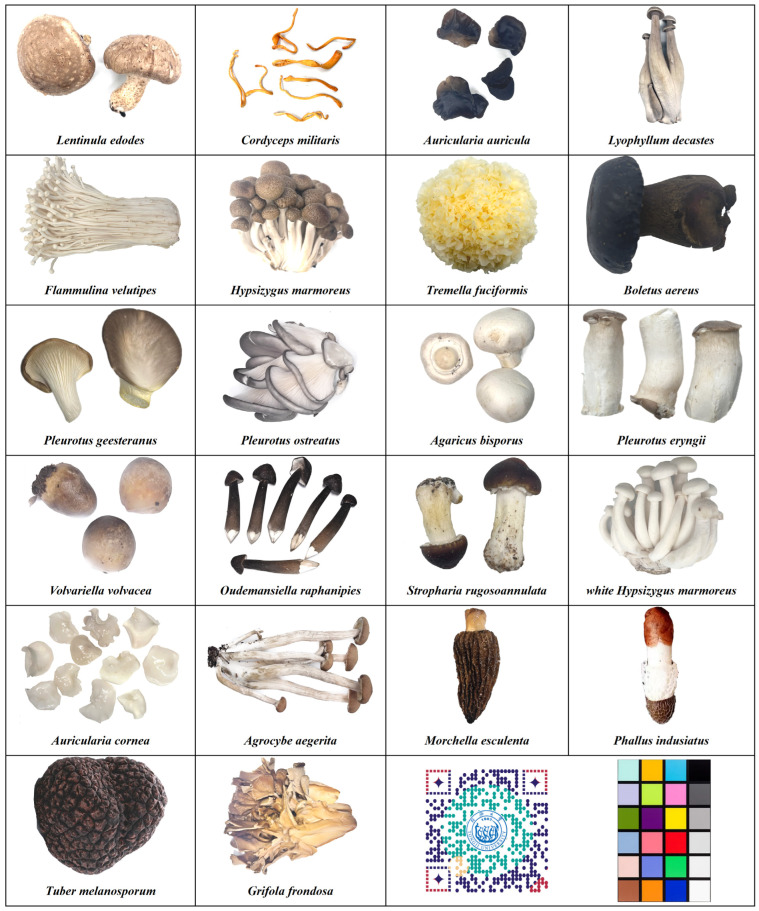
Twenty-two varieties of fresh cultivated edible mushrooms and edible wild mushrooms commonly found in the Chinese market.

In the regular consumer market for mushrooms in China (including supermarkets, large shopping malls, agricultural product wholesale centers, etc.), as long as the Chinese and scientific names of cultivated edible mushrooms are accurate, common fresh edible mushroom products (Figure 3) such as *L. edodes*, white *Hypsizygus marmoreus*, *Hypsizygus marmoreus*, *F. velutipes*, *Tremella fuciformis*, *P. eryngii*, *Pleurotus geesteranus*, *P. ostreatus*, *A. bisporus*, *O. raphanipies*, *Lyophyllum decastes*, *Volvariella volvacea*, *Auricularia cornea*, *A. auricula*, *Agrocybe aegerita*, *Phallus indusiatus*, *Grifola frondosa*, *C. militaris*, *Morchella esculenta*, and *S. rugosoannulata* are unlikely to be counterfeited. Simultaneously, there is a relatively high risk of poisoning from consuming wild mushrooms, and varieties of commercially available edible wild mushrooms are limited. Currently, edible wild mushrooms such as *Tuber melanosporum* and *Boletus aereus* are readily available on the market (Figure 3). By mandating sellers to accurately use standardized Chinese and scientific names for edible mushrooms and prohibiting incorrect names or species abbreviations, the issue of misleading consumers into purchasing incorrectly labeled mushroom products can be effectively addressed.

However, as discussed in “3. The reasons for the phenomenon of selling fake edible mushrooms”, counterfeit or low-quality edible mushrooms are found in trading venues (Table 1). When consumers purchase edible mushroom products—especially wild mushrooms and cultivated mushrooms with medicinal value—from informal sources (such as roadside stalls, illegal shops near tourist attractions, etc.), it becomes easy for sellers to distribute counterfeit products [16,23,29]. Additionally, when whole, sliced, or powdered edible fungus products are sold online and offline, both domestically and internationally, counterfeit products may also circulate [19,20,22]. To safeguard the legitimate rights and interests of consumers and facilitate market regulatory authorities to identify counterfeit products, it is essential to develop reliable technologies capable of identifying fake mushroom products.

Currently, researchers employ various methods, such as Fourier transform near-infrared spectroscopy with the random forest method, the gas chromatography–mass spectrometry method, the two-dimensional infrared correlation spectroscopy method, the polymerase chain reaction-restriction fragment length polymorphism method, the real-time quantitative PCR method, dual-channeled capillary electrophoresis technology, the two-dimensional gel electrophoresis method, the HPLC fingerprint method, the surface-enhanced laser Raman spectroscopy method, MALDI-ToF mass spectrometry technology, and near-infrared spectral analysis technology, for the authentication of counterfeit mushroom products [15,16,17,18,19,20,21,22,23,25,27,30].

**Figure 4:**
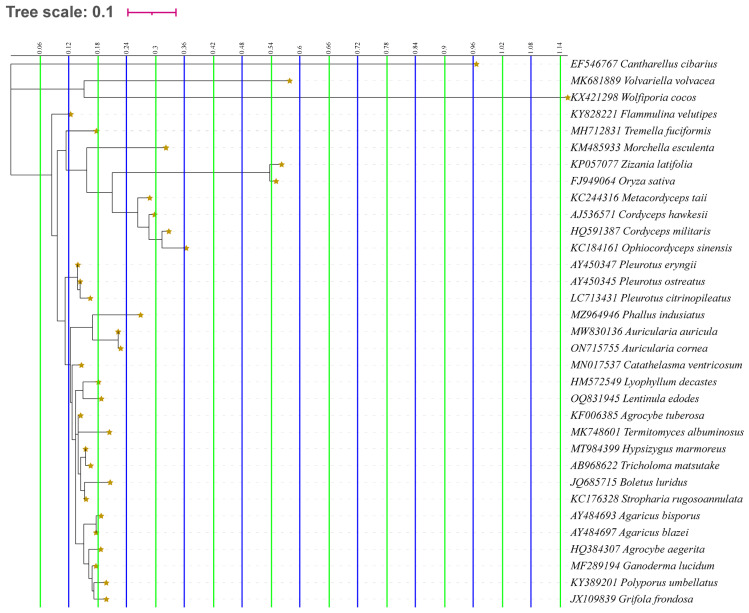
ML phylogenetic tree constructed using standard partial ITS sequences from 33 species.

Taking DNA barcode technology as an example, it is currently a research scheme with minimal sample loss, a broad applicability for identification, a simple operational method, and low experimental costs [31]. However, it has not been widely used in the identification of counterfeit mushroom products. Utilizing the NCBI database (https://www.ncbi.nlm.nih.gov/, accessed on 31 August 2024), we obtained the ITS (Internal transcribed spacer) sequences of all species mentioned in Table 1, along with common edible fungus species on the mushroom consumer market. Although we were unable to acquire the ITS sequences of *B. purpureus*, *O. raphanipies*, *S. geobombycis*, and *P. geesteranus*, most species’ ITS sequences can be accessed from the database (Table S3). A maximum likelihood (ML) phylogenetic tree was constructed, with bootstrap supports calculated from 1000 replicates [31]. The ML phylogenetic tree results depicted in Figure 4 revealed significant genetic distances among the ITS sequences of 33 species, enabling effective differentiation among samples. This suggests that, after obtaining the ITS sequences of mushroom samples for identification, their species names can be determined through comparison using the NCBI’s BLAST^®^ system (https://blast.ncbi.nlm.nih.gov/Blast.cgi?PROGRAM=blastn&PAGE_TYPE=BlastSearch&LINK_LOC=blasthome, accessed on 31 August 2024). The extensive application of this technology has the potential to mitigate fraudulent practices and provide a robust technical solution for establishing an edible fungi identification system.

Nevertheless, a limitation is that the NCBI database still lacks ITS standard sequences for some mushroom species, which should be further supplemented in the future. Regarding the identification of individual species, it is important to note that obtaining standardized and highly accurate DNA barcode sequences requires collaboration between expert taxonomists and molecular biologists. These sequences should ultimately be uploaded to genetic databases, such as the NCBI database, to provide reliable DNA barcode reference sequences for other researchers. This makes species identification more objective and reliable. Researchers must have a fundamental understanding of both morphological and molecular identification to prevent flawed data from affecting the reliability and accuracy of public databases. Additionally, researchers should pay attention to several factors during the DNA barcode acquisition process [32,33,34,35,36], as follows:

(1) Collecting healthy, fresh samples for DNA barcode research, as damaged DNA can lead to incorrect sequencing data;
(2) Considering primer specificity, as inappropriate primers may cause base mismatches or the misidentification of species;
(3) Not using gene sequences with overlapping peaks, as the absence of rigorous quality control in molecular experiments might give rise to the overlapping of multiple peaks during sequencing, which can directly exert an influence on the accuracy and readability of the sequencing outcomes;
(4) Manually checking and correcting sequences that are free from overlapping peaks with strict accuracy;
(5) Proficiently using genetic databases and comparison techniques to avoid errors in species identification due to improper methods;
(6) Strictly overseeing and reviewing data to ensure that the DNA barcode sequences uploaded to genetic databases are accurate and reliable.

Meanwhile, criteria for evaluating mixed samples need to be established. It is essential to develop new technologies that exhibit a broad applicability and stable performance. For instance, environmental DNA metagenomic technology is gradually being used to identify mixed samples [37,38]. Unfortunately, this technology has not yet been applied in the identification of edible fungi. Therefore, developing DNA barcoding or DNA metagenomic technology specifically for identifying counterfeit mushroom products or mixed mushroom samples holds significant application potential. However, it should be noted that relying solely on molecular biology methods may not accurately identify the specific part of the edible fungi from which the product originates. For instance, these methods cannot distinguish between *L. edodes* pileus powder (a high-quality product) and *L. edodes* stipe powder (a low-quality product). Consequently, it is necessary to integrate techniques such as HPLC fingerprinting to further differentiate counterfeit or low-quality edible fungi products [22]. In the near future, a combination of various technologies is likely to result in the establishment of an effective system for identifying edible fungi.

### 4.2. Caution Is Required When Consuming Wild Mushrooms

**Figure 5:**
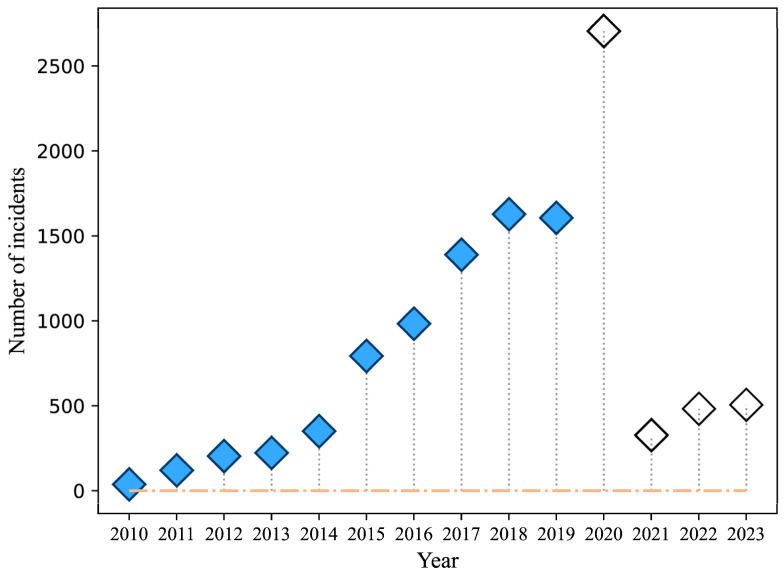
The number of incidents reported mushroom poisoning outbreaks in the Chinese mainland from 2010 to 2023. Notes: “
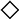
” means the years affected by the COVID-19 pandemic, whereas the remaining years remain unimpacted by the pandemic.

While some edible fungi (non-toxic or lowly toxic) can be found among wild mushrooms, many species, such as those in the *Amanita* genus [13,21], are highly toxic. Wild mushroom products served in catering establishments (street stalls, staff canteens, restaurants, rural banquets, cafes, fast food stores, etc.) are often contaminated with poisonous varieties. On the Chinese mainland, there were 11,350 reported incidents of mushroom poisoning outbreaks from 2010 to 2023. This figure exhibited a gradual increase from 2010 to 2019, followed by an exponential surge in 2020, a significant decrease in 2021, and a subsequent return to pre-2015 levels. From 2021 to 2023, the annual number of incidents stabilized at a low level (Figure 5; Table S4).

The total number of affected patients involved from 2010 to 2023 reached 42,234, as shown in Table S4. Unfortunately, there were 852 deaths, resulting in a mortality rate of 2.02% (Table S4). Symptoms of poisoning (nausea, vomiting, abdominal pain, diarrhea, dizziness, headache, fever, etc.) were primarily linked to the following toxic wild mushroom species: *Chlorophyllum molybdites*, *Russula japonica*, *Scleroderma cepa*, *Gymnopilus dilepis*, *Boletus bainiugan*, *Amanita exitialis*, *Amanita fuliginea*, *Amanita rimosa*, *Galerina sulciceps*, *Russula subnigricans*, *Lepiota brunneoincarnata*, *Omphalotus* sp., *Russula pseudojaponica*, *Amanita subglobosa*, and *Entoloma omiense*. The species primarily responsible for fatalities included *A. exitialis*, *Amanita subpallidorosea*, *R. subnigricans*, *G. sulciceps*, and *A. rimosa* [10,11,12,13,14].

The regions on the Chinese mainland with the top 20% of reported incidents of mushroom poisoning outbreaks from 2010 to 2023 were Yunnan Province (4281 incidents, 37.72%), Hunan Province (1952 incidents, 17.20%), Guizhou Province (1443 incidents, 12.71%), Sichuan Province (774 incidents, 6.82%), Jiangxi Province (364 incidents, 3.21%), Fujian Province (280 incidents, 2.47%), and Guangxi Zhuang Autonomous Region (276 incidents, 2.43%) (Table S5). Notably, 2020 witnessed a record high of 2705 reported mushroom poisoning incidents, likely influenced by factors such as the COVID-19 pandemic and a limited food supply during prevention measures, which increased the risk of the accidental consumption of poisonous wild mushrooms. The subsequent decline in incidents in 2021, 2022, and 2023 (Figure 5) may be attributed to extensive public education efforts by the Chinese government aimed at preventing wild mushroom poisoning. In summary, while wild mushrooms are celebrated for their unique flavors and are consumed in many countries [21,24,26], enforcing a complete ban on their consumption is impractical as a preventive measure against food poisoning. Therefore, individuals should avoid consuming unfamiliar wild mushrooms unless they possess adequate knowledge to distinguish safe species or purchase non-toxic varieties certified by regulatory authorities.

### 4.3. Considerations for the Future Development of the Edible Fungi Industry

The edible mushroom industry plays a crucial role in poverty alleviation, and globally, edible mushrooms are regarded as a luxury food item. With recent challenges posed by COVID-19 and regional droughts and floods [39,40,41], the production and consumption market for edible mushrooms has shown a temporary contraction. However, a positive long-term development trend is anticipated [42]. Since 2023, the edible mushroom industry has been gradually recovering. Future development should focus on the following four points:

Firstly, it is essential to advance the cultivation of edible fungi, providing Chinese expertise and solutions for international poverty alleviation initiatives. Increased investment in the research and development of high-quality end products is vital, targeting breakthroughs in volatile compound extraction, the purification of anti-cancer active substances, and enzyme product development. Integrating research concepts from other industries [43,44,45] will also enhance the deep processing of edible fungi products.

Secondly, efforts must be intensified to combat the commercialization of counterfeit and mislabeled edible mushroom products to uphold product quality and restore market confidence in China’s position as the world’s leading producer. This includes developing technical methods for regulatory authorities to identify counterfeit or low-quality products (e.g., the gas chromatography–mass spectrometry method, the real-time quantitative PCR method, DNA barcoding identification technology, the HPLC fingerprint method, the surface-enhanced laser Raman spectroscopy method, MALDI-ToF mass spectrometry technology, and near-infrared spectral analysis technology) [15,16,17,18,21,22,27,46]. Additionally, public service electronic manuals should be created to help consumers to identify fraudulent edible mushroom products.

Thirdly, enhancing public awareness is crucial for preventing the accidental consumption of poisonous wild mushrooms, particularly in the regions of southwest and southeast China, where incidents of mushroom poisoning are prevalent (Table S4). Frequent poisoning occurrences may deter consumer interest in edible mushrooms, hindering the long-term development of the edible mushroom industry. Regulatory authorities should focus on accurately identifying toxic species and preventing their sale in food service establishments, alongside sustained educational efforts to improve the public recognition of poisonous varieties.

Lastly, there is an urgent need to advance artificial cultivation technologies for edible wild mushrooms. Varieties such as *T. melanosporum*, *T. albuminosus*, *Boletus tomentipes*, *B. purpureus*, *B. aereus*, *Boletus albus*, *Boletus umbriniporus*, *B. bainiugan*, *Rugiboletus extremiorientalis*, and *O. sinensis* possess significant market value and various edible and medicinal properties [23]. However, they are currently only available in natural habitats. Achieving the artificial cultivation of high-value edible wild mushrooms would not only reduce the risk of accidental consumption of poisonous varieties, but also provide substantial economic benefits and advance the edible fungus industry in the foreseeable future.

## Figures and Tables

**Table 1:** Detailed information on the current counterfeit or low-quality status of edible mushrooms.

Name of Genuine Raw Material(s)	The Main Use Value of Genuine Raw Material(s)	Form of Product	Name of Counterfeit Raw Material(s)	The Main Form of Product Transaction	Reference(s)
*Lentinula edodes* pileus powder	Food industry, pharmaceutical industry, and daily chemical industry	Powder	*Lentinula edodes* stipe powder, *Pleurotus eryngii* powder, or *Pleurotus ostreatus* powder	Global trade and local trade	[22]
*Wolfiporia cocos*	Pharmaceutical industry	Fruiting body	Substitution with rice (*Oryza sativa*) powder	Local trade	[15]
*Polyporus umbellatus*	Pharmaceutical industry	Sclerotium	Alum solution, *Agrocybe tuberosa*	Local trade	[19,24]
*Tricholoma matsutake*	Food industry and pharmaceutical industry	Fruiting body	*Stropharia rugosoannulata*, *Pleurotus eryngii*, *Catathelasma ventricosum*, or *Agaricus blazei*	Global trade and local trade	[18]
*Ganoderma lucidum* spore powder	Pharmaceutical industry, food industry, and daily chemical industry	Powder	Use of dried starch as an adulterant	Global trade and local trade	[25]
*Cantharellus cibarius*	Food industry and pharmaceutical industry	Fruiting body	*Pleurotus citrinopileatus* or *Zizania latifolia*	Local trade	[26]
*Termitomyces albuminosus*	Food industry and pharmaceutical industry	Fruiting body	*Oudemansiella raphanipies*	Local trade	[27]
*Boletus purpureus*	Food industry	Fruiting body	*Boletus luridus*	Local trade	[28]
*Ophiocordyceps sinensis*	Food industry, pharmaceutical industry, and daily chemical industry	Fruiting body	*Cordyceps militaris*, *Cordyceps hawkesii*, *Metacordyceps taii*, dough, or the root of the *Stachys geobombycis*	Global trade and local trade	[29,30]

## Data Availability

The original contributions presented in the study are included in the article and Supplementary Materials, further inquiries can be directed to the corresponding authors.

## Supplementary Materials

The following supporting information can be downloaded at: https://www.mdpi.com/article/10.3390/foods13193097/s1, Table S1: Production of edible mushrooms in various countries or regions in 2022; Table S2: Production of edible mushrooms across provinces, autonomous regions, municipalities, and special administrative regions of China in 2022; Table S3: Genetic sequence information used in this study after sequence editing; Table S4: Number of incidents, patients, and deaths in mushroom poisoning outbreaks reported in Mainland China from 2010 to 2023; Table S5: Number of incidents in various provinces, autonomous regions, and municipalities in Mainland China from 2010 to 2023.

## References

[1] Wang X.M., Zhang J., Wu L.H., Zhao Y.L., Li T., Li J.Q., Wang Y.Z., Liu H.G. (2014). A mini-review of chemical composition and nutritional value of edible wild-grown mushroom from China. Food Chem..

[2] Ahmad I., Arif M., Xu M.M., Zhang J.Y., Ding Y.T., Lyu F. (2023). Therapeutic values and nutraceutical properties of shiitake mushroom (*Lentinula edodes*): A review. Trends Food Sci. Technol..

[3] FAOSTAT (2024). Mushroom & Trufes: Producer Prices. https://www.fao.org/faostat/en/#data/PP/.

[4] Niego A.G.T., Lambert C., Mortimer P., Thongklang N., Rapior S., Grosse M., Schrey H., Charria-Girón E., Walker A., Hyde K.D. (2023). The contribution of fungi to the global economy. Fungal Divers..

[5] Liu F.S., Lin D.G., Lin H., Liu H.W., Lin Z.X. (2017). Physiological and photosynthetic responses of giant juncao (*Pennisetum giganteum*) to drought stress. Fresenius Environ. Bull..

[6] Zhou J., Chen S.Q., Shi W.J., David-Schwartz R., Li S.T., Yang F.L., Lin Z.X. (2021). Transcriptome profiling reveals the effects of drought tolerance in Giant Juncao. BMC Plant Biol..

[7] Chang S.T., Buswell J.A. (2008). Development of the world mushroom industry: Applied mushroom biology and international mushroom organizations. Int. J. Med. Mushrooms.

[8] Xu F., Gao S., Yang J.X., Yang L.Y., Huo Q.L., Wang Q. (2021). Application of HACCP system in quality control of *Auricularia auricula* in main production area. J. Food Saf. Qual..

[9] Tan Q. (2017). The Development of Xianggu Industry in China.

[10] Li H.J., Zhang H.S., Zhang Y.Z., Zhang K.P., Zhou J., Yin Y., Jiang S.F., Ma P.B., He Q., Zhang Y.T. (2020). Mushroom Poisoning Outbreaks—China, 2019. China CDC Wkly..

[11] Li W.W., Pires S.M., Liu Z.T., Liang J.J., Wang Y.F., Chen W., Liu C.W., Liu J.K., Han H.H., Fu P. (2021). Mushroom Poisoning Outbreaks—China, 2010–2020. China CDC Wkly..

[12] Li H.J., Zhang H.S., Zhang Y.Z., Zhou J., Yin Y., He Q., Jiang S.F., Ma P.B., Zhang Y.T., Yuan Y. (2022). Mushroom Poisoning Outbreaks—China, 2021. China CDC Wkly..

[13] Li H.J., Zhang Y.Z., Zhang H.S., Zhou J., Liang J.Q., Yin Y., He Q., Jiang S.F., Zhang Y.T., Yuan Y. (2023). Mushroom Poisoning Outbreaks—China, 2022. China CDC Wkly..

[14] Li H.J., Zhang Y.Z., Zhang H.S., Zhou J., Chen Z.H., Liang J.Q., Yin Y., He Q., Jiang S.F., Zhang Y.T. (2024). Mushroom Poisoning Outbreaks—China, 2023. China CDC Wkly..

[15] Zhou X., Zhang Y.S., Zhao Y., Gong X.J., Zhao C., Chen H.G. (2009). An LC Fingerprint Study of *Poria cocos* (Schw.) Wolf. Chromatographia.

[16] Zhang W.J., Zhang X.L., Li M.H., Shi Y., Zhang P., Cheng X.L., Wei F., Ma S.C. (2017). Identification of Chinese caterpillar medicinal mushroom, *Ophiocordyceps sinensis* (Ascomycetes), from counterfeit species. Int. J. Med. Mushrooms.

[17] Zhang H.Y., Li Y.H., Mi J.N., Zhang M., Wang Y.R., Jiang Z.H., Hu P. (2017). GC-MS profiling of volatile components in different fermentation products of *Cordyceps sinensis* mycelia. Molecules.

[18] Li Y.Q., Pan T.H., Li H.R., Zou X.B. (2019). NIR spectral feature selection using lasso method and its application in the classification analysis. Spectrosc. Spectr. Anal..

[19] Chen X.D., Choong Y., Zhang W.W., Li G.Q., Lan J. (2020). Discrimination of authentic *Polyporus umbellatus* and counterfeit by Fourier Transform Infrared and two dimensional infrared correlation spectroscopy. J. Mol. Struct..

[20] Nguyen T.D., Vu M.T., Nguyen M.H., Duong H.A., Mai T.D., Pham H.V. (2021). A Rapid and Simple Dual-Channeled Capillary Electrophoresis with Contactless Conductivity Detection Method for the Determination of Adenosine, Cordycepin, and Inosine in *Ophiocordyceps sinensis*-Based Products. Food Anal. Methods.

[21] Piarroux R., Gabriel F., Grenouillet F., Collombon P., Louasse P., Piarroux M., Normand A.C. (2021). Using MALDI-ToF mass spectrometry to identify mushroom species: Proof of concept analysis of *Amanita* genus specimens. Med. Mycol..

[22] Guo Y.H., Sun K.X., Cheng Y.L., Yu H., Xie Y.F., Zhang H.W., Yao W.R., Qian H. (2022). Authentication of shiitake powder using HPLC fingerprints combined with chemometrics. Eur. Food Res. Technol..

[23] Yan Z.Y., Liu H.G., Zhang S., Li J.Q., Wang Y.Z. (2022). Superiority of two-dimensional correlation spectroscopy combined with ResNet in species identification of bolete. Infrared Phys. Technol..

[24] Zhao Y.Y. (2013). Traditional uses, phytochemistry, pharmacology, pharmacokinetics and quality control of *Polyporus umbellatus* (Pers.) Fries: A review. J. Ethnopharmacol..

[25] Shi X.Y., Gan X.Q., Wang X.B., Peng J.L., Li Z.H., Wu X.Q., Shao Q.S., Zhang A.L. (2022). Rapid detection of *Ganoderma lucidum* spore powder adulterated with dyed starch by NIR spectroscopy and chemometrics. LWT-Food Sci. Technol..

[26] Dimopoulou M., Alexandros K., Stamatis M., Odysseas A., Olga G. (2022). Nutritional composition and biological properties of sixteen edible mushroom species. Appl. Sci..

[27] Liu Y., Li L., Wei Y.M., Zhang H.Y., Xiang S.N., Shang Y. (2022). A specific gene, *TSA*, used as endogenous reference gene for qualitative and real-time quantitative PCR detection of *Termitomyces albuminosus*. LWT-Food Sci. Technol..

[28] Falandysz J., Zhang J., Wang Y.Z., Saba M., Krasinska G., Wiejak A., Li T. (2015). Evaluation of Mercury Contamination in Fungi *Boletus* Species from Latosols, Lateritic Red Earths, and Red and Yellow Earths in the Circum-Pacific Mercuriferous Belt of Southwestern China. PLoS ONE.

[29] Hsu T.H., Shiao L.H., Hsieh C.Y., Chang D.M. (2002). A comparison of the chemical composition and bioactive ingredients of the Chinese medicinal mushroom DongChongXiaCao, its counterfeit and mimic, and fermented mycelium of *Cordyceps sinensis*. Food Chem..

[30] Ho-Chan R.C., Wo-Lam S.S., Yan-Fong F.L., Wah-Chan D.T., Fat-Lee F.W., Po-Sze E.T. (2018). Optimization of protein extraction and two-dimensional gel electrophoresis profiles for the identification of *Cordyceps sinensis* and other similar species. PLoS ONE.

[31] Liu J.L., Tong Y.C., Xia J., Sun Y.Q., Zhao X.H., Sun J.Y., Zhao S., Zhuang M.M., Zhang J.H., He P.M. (2022). *Ulva* macroalgae within local aquaculture ponds along the estuary of Dagu River, Jiaozhou Bay, Qingdao. Mar. Pollut. Bull..

[32] Chen L.X., Liu P.F., Evans T.C., Ettwiller L.M. (2017). DNA damage is a pervasive cause of sequencing errors, directly confounding variant identification. Science.

[33] Steinegger M., Salzberg S.L. (2020). Terminating contamination: Large-scale search identifies more than 2,000,000 contaminated entries in GenBank. Genome Biol..

[34] Li Q., Luo X.Y., Liu Q., Cheng Z.T., Huang X.L. (2022). Reducing human error to improve efficiency of DNA barcoding: The primer case. Zool. Syst..

[35] Tong Y.C., Xia L.H., Liu J.L., Zhao S., Sun Y.Q., Wu T.J., Xia Z.Y., Li S., Cao J.X., Zhang J.H. (2022). Distribution and Identification of *Ulva aragoensis* (Ulvaceae, Chlorophyta), a Constituent Species of Green Tides in the Southern Yellow Sea, Based on Molecular Data. J. Mar. Sci. Eng..

[36] Xia J., Hu H.J., Gao X., Kan J.J., Gao Y.H., Li J. (2024). Phytoplankton Diversity, Spatial Patterns, and Photosynthetic Characteristics Under Environmental Gradients and Anthropogenic Influence in the Pearl River Estuary. Biology.

[37] Zeng Y.Q., Wang X.R., Liu J.L., Cao J.X., Sun Y.Q., Zhao S., Chen Z.H., Kim J.K., Zhang J.H., He P.M. (2024). Harnessing the power of eDNA technology for macroalgal ecological studies: Recent advances, challenges, and future perspectives. Algal Res.-Biomass Biofuels Bioprod..

[38] Yun K.W., Son H.S., Seong M.J., Lee S.M., Kim M.C. (2024). Enhanced eDNA monitoring for detection of viable harmful algal bloom species using propidium monoazide. Harmful Algae.

[39] Kour H., Kour D., Kour S., Singh S., Hashmi S.A.J., Yadav A.N., Kumar K., Sharma Y.P., Ahluwalia A.S. (2022). Bioactive compounds from mushrooms: Emerging bioresources of food and nutraceuticals. Food Biosci..

[40] Liu Y., Li Y., Wang G.F., Gao G., Chen Y.X. (2023). Quantifying multi-regional indirect economic losses: An assessment based on the 2021 rainstorm events in China. Front. Earth Sci..

[41] Wang X.F., Luo P.P., Zheng Y., Duan W.L., Wang S.T., Zhu W., Zhang Y.Z., Nover D. (2023). Drought Disasters in China from 1991 to 2018: Analysis of Spatiotemporal Trends and Characteristics. Remote Sens..

[42] Cao B. (2023). Characteristics and trend of shiitake mushroom consumption market in China in the new era. Acta Edulis Fungi.

[43] Li S., Hu M.J., Tong Y.P., Xia Z.Y., Tong Y.C., Sun Y.Q., Cao J.X., Zhang J.H., Liu J.L., Zhao S. (2023). A review of volatile compounds in edible macroalgae. Food Res. Int..

[44] Sun Y.Q., Liu J.L., Xia J., Tong Y.C., Li C.X., Zhao S., Zhuang M.M., Zhao X.H., Zhang J.H., He P.M. (2022). Research development on resource utilization of green tide algae from the Southern Yellow Sea. Energy Rep..

[45] Huang W. (2017). Cooperative governance of e-commerce selling counterfeits under the new governance theory. Soc. Sci..

[46] Sun J.H., Feng Y., Wang S., Sheng Y., An L.P., Du P.G., Sun J.B. (2022). Identification of *Cordyceps sinensis* and *Cordyceps militaris* by surface enhanced Raman spectroscopy with chemometric analysis. Chin. J. Pharm. Anal..

