# Supplementary material for: The Situation of Counterfeited and Mislabeled Commercialized Edible Mushrooms in China and the Development of Possible Controls
Source: Foods. 2024 Sep 27;13(19):3097. doi: 10.3390/foods13193097 (PMC11476016; doi:10.3390/foods13193097)
Supplement: Supplementary file 1 [file foods-13-03097-s001.zip › Supplemental Information-2024.09.21.pdf]

SUPPLEMENTAL INFORMATION

Table S1. Production of edible mushrooms in various countries or regions in 2022.

**Country or region**	**Production (t)**
China, mainland	45428825
Japan	469492
United States of America	318600
India	280000
Poland	256800
Netherlands (Kingdom of the)	235000
Spain	167030
Canada	139090
Russian Federation	128704
France	101800
United Kingdom of Great Britain and Northern Ireland	84148
Germany	78160
Italy	67440
Ireland	65910
Tajikistan	65636
Indonesia	63155
Hungary	51050
Australia	50500
South Africa	32116
Belgium	28000
Iran (Islamic Republic of)	25628
Viet Nam	25081
Belarus	24056
Republic of Korea	23460
Israel	15000
Romania	14730
Portugal	13940
Ukraine	12198
China, Taiwan Province	9720
Lithuania	9250
Switzerland	8625
Democratic People's Republic of Korea	6037
Serbia	5480
North Macedonia	2945

Madagascar	2825
Greece	2560
Morocco	2220
Republic of Moldova	1939
Bulgaria	1630
Algeria	1584
Azerbaijan	1567
New Zealand	1448
Sweden	1380
Cyprus	1300
Bosnia and Herzegovina	1268
Finland	1070
Jordan	1001
Malta	920
Zimbabwe	893
Philippines	794
Uzbekistan	675
Montenegro	600
Iceland	592
Kazakhstan	515
Armenia	344
Mongolia	341
Kyrgyzstan	231
Tunisia	178
Singapore	174
Thailand	173
Albania	100
Kuwait	< 100
Brunei Darussalam	< 100
China, Hong Kong SAR	< 100

Notes: Data provided by the Food and Agriculture Organization of the United Nations (FAO).

Table S2. Production of edible mushrooms across provinces, autonomous regions, municipalities, and special administrative regions of China in 2022.

First-level local state administrative organ	Production (t)
Henan Province	6024600
Fujian Province	4891000
Heilongjiang Province	3889000
Hebei Province	3380400
Shandong Province	3013100
Sichuan Province	2447800
Jilin Province	2061000
Guizhou Province	1804900
Jiangsu Province	1547100
Hubei Province	1509200
Jiangxi Province	1494500
Liaoning Province	1386900
Shaanxi Province	1276400
Hunan Province	1201700
Guangxi Zhuang Autonomous Region	1042400
Yunnan Province	983300
Guangdong Province	892200
Anhui Province	707900
Zhejiang Province	678100
Inner Mongolia Autonomous Region	525700
Shanxi Province	451600
Chongqing Municipality	373900
Gansu Province	334700
Xinjiang Uygur Autonomous Region	103300
Tianjin Municipality	75000
Beijing Municipality	74200
Shanghai Municipality	40400
Taiwan Province	9720
Qinghai Province	7800
Xizang Tibetan Autonomous Region	7400
Hainan Province	No information
Ningxia Hui Autonomous Region	No information
Hong Kong Special Administrative Region	< 100
Macao Special Administrative Region	0

Notes: Statisticians from Hainan Province and Ningxia Hui Autonomous Region aggregate data for vegetables and edible mushrooms, but detailed classification data is unavailable. Data provided by the China Edible Fungi Association (CEFA) and FAO.

Table S3. Genetic sequence information used in this study after sequence editing.

Latin name of species	Accession number	Base sequence
Lentinula edodes	OQ831945	AACTTTCAGCAACGGATCTCTTGGCTCTCCCATCGATGAA<br>GAACGCAGCGAAATGCGATAAGTAATGTGAATTGCAGAA<br>TTCAGTGAATCATCGAATCTTTGAACGCACCTTGCGCCCT<br>CTGGTATTCCGGAGGGCATGCCTGTTTGAGTGTCATTAAA<br>TTCTCAACTTTATAAGTTTTTGCTTATTAAAGCTTGGATATT<br>GGAGGTTTGCAGGCGTTTTGTCAGCTCCTCTTAAATTTATT<br>AGTG
Agaricus blazei	AY484697	AACTTTCAGCAACGGATCTCTTGGCTCTCGCATCGATGAA<br>GAACGCAGCGAAATGCGATAAGTAATGTGAATTGCAGAA<br>TTCAGTGAATCATCGAATCTTTGAACGCATCTTGCGCTCC<br>TTGGTATTCCGAGGAGCATGCCTGTTTGAGTGTCATTAAA<br>TTCTCAACTCTCTTATACTTTTTTGTAAAAGAGAGCTTGG<br>ACTGTGGAGGCTTGCTGGCCACTTTTTGGGGTCAGCTCCT<br>CTGAAATGCATTAGCG
Cantharellus cibarius	EF546767	GTCCGCAGGCGGCGGGATGACTTGGGTTGGAAGAGGGA<br>AGGAGAAGGGGACTGGGTTAGCATAGGCGGCGTTGCCA<br>GTGCGCGCTGCTGAGGAGCAACCGGCCAGTTGGTCCGC<br>GCTACTTGGTCGATCTCTCTGCTGGACTTGTTGGGTGTAA<br>GGTAGGCATCCACGGTTATTTTGGCGTGCACCCTCCTTTT<br>TGGCGTGCACCCTCCTTCCGTTGTGCCCAATCGTCTGAAG<br>TTATGGGCCTCGACGAATTGATGTAAGTCGAGGGGGTCAT<br>ATCTGTTTTTGGCCCGGCATGG
Ophiocordyceps sinensis	KC184161	AACTTTCAACAACGGATCTCTTGGTTCTGGCATCGATGAA<br>GAACGCAGCGAAATGCGATAAGTAATGTGAATTGCAGAA<br>TTCAGTGAACCATCGAATCTTTGAACGCACATTGCGCCC<br>GCCAGCACTCTGGCGGGCATGCCTGTCCGAGCGTCATCT<br>CAACCCTCGAGCCCCCCGCCTCGCGGCGGCGGGGCCCG<br>GCCTTGGGGGTCACGGCCCCGCGCCGCCCCCTAAACGCA<br>GTGGCG
Ganoderma lucidum	MF289194	AACTTTCAGCAACGGATCTCTTGGCTCTCGCATCGATGAA<br>GAACGCAGCGAAATGCGATAAGTAATGTGAATTGCAGAA<br>TTCAGTGAATCATCGAATCTTTGAACGCACCTTGCGCTCC<br>TTGGTATTCCGAGGAGCATGCCTGTTTGAGTGTCATGAAA<br>TCTTCAACCTGCAAGCTTTTGTGGTTTGTAGGCTTGGACT<br>TGGAGGCTTGTCGGCCGTTGTTGGTCGGCTCCTCTTAAAT<br>GCATTAGCT
Pleurotus citrinopileatus	LC713431	AACTTTCAACAACGGATCTCTTGGCTCTCGCATCGATGAA<br>GAACGCAGCGAAATGCGATAAGTAATGTGAATTGCAGAA<br>TTCAGTGAATCATCGAATCTTTGAACGCACCTTGCGCCCC<br>TTGGTATTCCGAGGGGCATGCCTGTTTGAGTGTCATTAAA<br>TTCTCAAACCTACCTTTTGCTTTGCTGTAAATCGTAGTGTT<br>TGGATTGTTGGGGGTTGCTGGCTTGTCACCGAGTCGGCTC<br>CTCTTAAATGCATTAGCG
Pleurotus	AY450347	AACTTTCAACAACGGATCTCTTGGCTCTCGCATCGATGAA

eryngii		GAACGCAGCGAAATGCGATAAGTAATGTGAATTGCAGAA<br>TTCAGTGAATCATCGAATCTTTGAACGCACCTTGCGCCCC<br>TTGGTATTCCGAGGGGCATGCCTGTTTGAGTGTCATTAAA<br>TTCTCAAACTCACTCTGGTTTTTCCAATTGTGATGTTTGGA<br>TTGTTGGAGGCTGCTGGCCTTGACAGGTCGGCTCCTCTTA<br>AATGCATTAGCA
Pleurotus ostreatus	AY450345	AACTTTCAACAACGGATCTCTTGGCTCTCGCATCGATGAA<br>GAACGCAGCGAAATGCGATAAGTAATGTGAATTGCAGAA<br>TTCAGTGAATCATCGAATCTTTGAACGCACCTTGCGCCCC<br>TTGGTATTCCGAGGGGCATGCCTGTTTGAGTGTCATTAAA<br>TTCTCAAACTCACTTTGGTTTTTTCCAATTGTGATGTTTGG<br>ATTGTTGGGGGCTGCTGGCCTTGACAGGTCGGCTCCTCTT<br>AAATGCATTAGCA
Wolfiporia cocos	KX421298	GCCGTCTAAGACCCGCTTGGCTTGACCTGTTGCACCGTCT<br>ACAGCCATCTTCCGAGTGTGTGCAATGGGAGAGAACGA<br>AGCCCGCGATTGGGGAATTCGAGATGCCCTCCCATTTATT<br>GGGGCGTGGGGAGGTTTGTGTACTCCCAGACCAGCTCCG<br>AGTCGTGCCGCCGTCTACTACCACAGACCTTTGTCTGAG<br>ACCGCTGGCCGATGCCGTCGAGCACGTCACAAGTCATCC<br>TCGAATTCACATCCGTCCGTCTATGACGGGCGCGGCC
Stropharia rugosoannula ta	KC176328	AACTTTCAGCAACGGATCTCTTGGCTCTCGCATCGATGAA<br>GAACGCAGCGAAATGCGATAAGTAATGTGAATTGCAGAA<br>TTCAGTGAATCATCGAATCTTTGAACGCACCTTGCGCTCC<br>TTGGTATTCCGAGGAGCATGCCTGTTTGAGTGTCATTAAA<br>TTCTCAACCTTTATCAGCTTTTTGGTTGATAAATGGCTTGG<br>ATGTGGGAGCTTGCAGGTTTCTCTTTTGAAATCAGCTCTC<br>CTGAAATACATTAGCT
Tricholoma matsutake	AB968622	AACTTTCAACAACGGATCTCTTGGCTCTCGCATCGATGAA<br>GAACGCAGCGAAATGCGATAAGTAATGTGAATTGCAGAA<br>TTCAGTGAATCATCGAATCTTTGAACGCACCTTGCGCTCC<br>TTGGTATTCCGAGGAGCATGCCTGTTTGAGTGTCATGAAA<br>TTCTCAACCTTTTCAGCTTTTTGTTGAATAGGCTTGGATTT<br>TGGGAGTTGTTGCAGGCTGCTCAGAAGTCTGCTCTCCTTA<br>AATGTATTAGCG
Cordyceps militaris	HQ591387	AACTTTCAACAACGGATCTCTTGGCTCTGGCATCGATGA<br>AGAACGCAGCGAAATGCGATAAGTAATGTGAATTGCAG<br>AATTCAGTGAATCATCGAATCTTTGAACGCACATTGCGCC<br>CGCCAGCATTCTGGCGGGCATGCCTGTTCGAGCGTCATTT<br>CAACCCTCGACGTCCCCTGGGGGATGTCGGCGTTGGGGA<br>CCGGCAGCACACCGCCGCCCCCGAAATGAAGTGGCG
Metacordycep s taii	KC244316	AACTTTCAACAACGGATCTCTTGGTTCTGGCATCGATGAA<br>GAACGCAGCGAAATGCGATAAGTAATGTGAATTGCAGAA<br>TTCAGTGAATCATCGAATCTTTGAACGCACATTGCGCCCG<br>TCAGTATTCTGGCGGGCATGCCTGTTCGAGCGTCATTACG<br>CCCCTCAAGTCCCCTGTGGACTTGGTGTTGGGGATCGGC<br>GAGGCTGGTTTTCCAGCRCAGCCGTCCCTYAAATYRATTG<br>GCG
Hypsizygus	MT984399	AACTTTCAACAACGGATCTCTTGGCTCTCGCATCGATGAA

marmoreus		GAACGCAGCGAAATGCGATAAGTAATGTGAATTGCAGAATTCAGTGAATCATCGAATCTTTGAACGCACCTTGCGCTCCTTGGTATTCCGAGGAGCATGCCTGTTTGAGTGTCATTAAATTCTCAACCTTTCCAGCTTTTATTAGCTTGGTCAGGCTTGGATGTGGGGGTTGCGGGCTTCTCAGAAGTCGGCTCTCCTTAAATGCATTAGCG
Flammulina velutipes	KY828221	AACTTTCAACAACGGATCTCTTGGCTCTCGCATCGATGAAGAACGCAGCGAAATGCGATAACTAATGTGAATTGCAGAATTCAGTGAATCATCGAGTCTTTGAACGCACCTTGCGCCCTTTGGTACTCCGAAGGGCATGCCTGTTTGAGTGTCAGTAACTTCTCAACCTCCCTCACTTTGTTGTGAGCTGGCGGATTGGACGTGGGGGCTTGCTGGACCTTATCTTTGGGTTCAGCTCCCCTGAAATGCATTAGCA
Tremella fuciformis	MH712831	AACTTTCAACAACGGATCTCTTGGCTCTCGCATCGATGAAGAACGCAGCGAATTGCGAAAAGTAATGTGAATTGCAGAATTCAGTGAATCATCGAATCTTTGAACGCACCTTGCGCCTTTTGGTATTCCGAAAGGCATGCCTGTTTGAGTGTCATGTAGACTCAACCCCCCGGGTTTCTGACCCGGCGGTGTTGGATTTGGGCCCTGCCTCTCCYGGCTGGCCTTAAATGCGTTAGTG
Agaricus bisporus	AY484693	AACTTTCAGCAACGGATCTCTTGGCTCTCGCATCGATGAAGAACGCAGCGAAATGCGATAAGTAATGTGAATTGCAGAATTCAGTGAATCATCGAATCTTTGAACGCATCTTGCGCTCCTTGGTATTCCGAGGAGCATGCCTGTTTGAGTGTCATTATATTCTCAACTCTCCAATACTTTGTTGTAAAGGAGAGCTTGGATTGTGGAGGTTTGCTGGCTCCTTACTTGGGGTCAGCTCCTCTGAAATGCATTAGCG
Lyophyllum decastes	HM572549	AACTTTCAGCAACGGATCTCTTGGCTCTCGCATCGATGAAGAACGCAGCGAAATGCGATAAGTAATGTGAATTGCAGAATTCAGTGAATCATCGAATCTTTGAACGCACCTGGCGCTCCCTGGTATTCCGGGGAGCATGTCTGTTTGAGTGTCATTAAATTCTCAACCTTTCCAACTTTTGCGAGTTTGGTTAGGCTTGGATGTGGAGGTTGCGGGCTTCACAGAAGTCGGCTCCTCTGAAATGCATTAGTG
Auricularia cornea	ON715755	AACTTTCAACAACGGATCTCTTGGCTCTCGCATCGATGAAGAACGCAGCGAAATGCGATAAGTAATGTGAATTGCAGAATTCAGTGAATCATCGAATCTTTGAACGCATCTTGCGCTCCTTGGTATTCCATGGAGCATGCCTGTTTGAGTGTCACGTAAACCCTCACCCTTGCGATGTAACAGTCGCTCGTGGTGGACTTGGACTGTGCCGTAACCGGCTCGTCTTGAAATGCATTAGCT
Grifola frondosa	JX109839	AACTTTCAGCAACGGATCTCTTGGCTCTCGCATCGATGAAGAACGCAGCGAAATGCGATAAGTAATGTGAATTGCAGAATTCAGTGAATCATCGAATCTTTGAACGCACCTTGCGCTCCTTGGTATTCCGAGGAGCATGCCTGTTTGAGTGTCATGGAATTCTCAACCCACACATCCTTGTGATGTGGACGGGCTTGGACTTTGGAGGCTCATGCCGGTCCCCATTCGGGTCGGCTCCTCTGGAATGCATTAGCT

Morchella esculenta	KM485933	AACTTTCAACAACGGATCTTCTTGGTTCCCACATCGATGAAGAACGCAGCGAAATGCGATAAGTAATGTGAATTGCAGAATTCAGTGAATCATCGAATCTTTGAACGCACATTGCGCCCTCTGGTATTCCGGAGGGCATGCCTGTTCGAGCGTCATAAATACCGCTCCCCCTCGGGATTGCTTGCGATCCCTGGGGGGTTCTGGCAATGTGGTCTCCCCGTGCTTTGAGGGCATGCGAACGGGCTTCCCAGTGCTGAAAGACATA
Phallus indusiatus	MZ964946	AACTTTCAACAACGGATCTCTTGGCTTTCGCATCGATGAAGAACGCCGCGAACGCGCGAAACGTAATGTGAATTGCAGAATTCAGTGAATCATCGAATCTTTGAACGCACCTTGCGCTCCTCGGTATTCCGAGGAGCATGCCTGTTTGAGTGTCGTGAAGTCTTATCGAGAAAGGGGGTCCCCTCGCGGGGGGGTCTCTTTTTTCGGACTTGGACGGTCTTGCCCCCCTCCCTCGTCCACAAGGGGGGGCTCGTCTTCAAATTTATCGGCG
Auricularia auricula	MW830136	AACTTTCAACAACGGATCTCTTGGCTCTCGCATCGATGAAGAACGCAGCGAAATGCGATAAGTAATGTGAATTGCAGAATTCAGTGAATCATCGAATCTTTGAACGCACCTTGCGCTCCTTGGTATTCCATGGAGCATGCCTGTTTGAGTGTCACGTAAACCCTCACCCCTGCGATGTAACAGTCGCCTGCGGTGGACTTGGACCGTGCCGGTAATCGGCTCGTCTTGAAATGCATTAGCT
Volvariella volvacea	MK681889	ATATTTTCACAAACGATGTTTTGGTCTCATCATCGTTATTAATGCCACTAAAATGCATAAAGAATGTTAAATGGCAAATTTCATGGATCCCCATACTTTAAAACCCCACTGGCGTTCTTGGGCAATTGTAAAAAGCTGACTTCTTTAATTGTCTCATATCTCAAGGCCCGTCCCGCTTCTCCCCCGGCTTTTGGGGGTTTGGGATTGGGAGACGGGGGGTTCCAATAGCTTCCAAATCACTTTCTTTAAGCATTCAGCAGGG
Oryza sativa	FJ949064	GACTCTCGGCAACGGATATCTCGGCTCTCGCATCGATGAAGAACGTAGCGAAATGCGATACCTGGTGTGAATTGCAGAATCCCGTGAACCATCGAGTCTTTGAACGCAAGTTGCGCCCGAGGCCATCCGGCCGAGGGCACGCCTGCCTGGGCGTCACGCCAAAAGACGCTCCACGCGCCCCCCCTATCCGGGAGGGCGCGGGGACGCGGTGTCTGGCCCCCCGCGCCTCGCGGCGCGGCGGGCCGAAGCTCGGGCTGCCG
Boletus luridus	JQ685715	AACTTTCAGCAACGGATCTCTTGGCTCTCGCATCGATGAAGAACGCAGCGAATTGCGATAAGTAATGTGAATTGCAGATTTTCAGTGAATCATCGAATCTTTGAACGCACCTTGCGCTCCTTGGTATTCCGAGGAGCATGCCTGTTTGAGTGTCATCGAATTCTCAACCATGTCTTGATCGATTTCAAGGCCATGGCTTGGAGTTGGGGGTTGCTGGCGGCGACGAGCTGTCGGCTCTCCTGAAATGCATTAGCA
Zizania latifolia	KP057077	GACTCTCGGCAACGGATATCTCGGCTCTCGCATCGATGAAGAACGCAGCGAAGTGCGATACCTGGTGTGAATTGCAGAATCCCGCGAACCATCGAGTCTTTGAACGCAAGTTGCGCCCGAGGCCATCCGGCCGAGGGCACGCCTGCCTGGGCGTCACGCCAAAAGACGCTCCGCGCCCCCCAGAGGCGCGGACGCGGTGTCTGGCCCCCCGTGCCGCGAGGCGCGGCGGG

		CCGAAGTTGGGGCTGCCG
Agrocybe aegerita	HQ384307	AACTTTCAGCAACGGATCTCTTGGCTCTCGCATCGATGAA<br>GAACGCAGCGAAATGCGATAAGTAATGTGAATTGCAAAA<br>TTCAGTGAATCATCGAATCTTTGAACGCACCTTGCGCTCC<br>TTGGTATTCCGAGGAGCATGCCTGTTTGAGTGTCATTACA<br>TTCTCAACCGTTTGAATTTTGCTATTTGAACGGCTTGGACT<br>TGGGGGTACTTTGTGCCGGCCCTAAAGGTCGGCTCCCCTT<br>AAATGCATTAGCT
Catathelasma ventricosum	MN01753<br>7	AACTTTCAACAACGGATCTCTTGGCTCTCGCATCGATGAA<br>GAACGCAGCGAAATGCGATAAGTAATGTGAATTGCAGAA<br>TTCAGTGAATCATCGAATCTTTGAACGCACCTTGCGCTCC<br>TTGGTATTCTGAGGAGCATGCCTGTTTGAGTGTCATTAAA<br>TTCTCAACCTTCTCCAGCTTTTGGAGTTGTGCTTGGATGT<br>GGGGGATGTGGGCTTCTCAGAAGTCAGCTCCTCTCAAAA<br>GCATTAGCA
Cordyceps hawkesii	AJ536571	AACTTTCAACAACGGATCTCTTGGTTCTGGCATCGATGAA<br>GAACGCAGCGAAATGCGATAAGTAATGTGAATTGCAGAA<br>TTCAGTGAATCATCGAATCTTTGAACGCACATTGCGCCCG<br>CCAGTACTCTGGCGGGCATGCCTGTTCGAGCGTCATTTCA<br>ACCCTCAGGCACCCCCCGCTGCGGCTGTGGCGGGCGGG<br>AGCCTGGTGTTGGGGACCGGCGGAAAACCTGCCCCCAG<br>GGCAGCCGCCGCCCCCTAAATGAATTGGCG
Polyporus umbellatus	KY389201	AACTTTCAGCAACGGATCTCTTGGCTCTCGCATCGATGAA<br>GAACGCAGCGAAATGCGATAAGTAATGTGAATTGCAGAA<br>TTCAGTGAATCATCGAATCTTTGAACGCACCTTGCGCTCC<br>TTGGTATTCCGAGGAGCATGCCTGTTTGAGTGTTGTGTAA<br>CTCTCAACCTGCAAACTTACTTGTAAATTTTGCAGGCTTG<br>GACTTTGGAGGCTTGTCGGCGCAAGTTGGCTCCTCTCAA<br>ATGCATTAGCT
Termitomyces albuminosus	MK748601	AACTTTCAGCAACGGATCTCTTGGCTCTCGCATCGATGAA<br>GGACGCAGCGAAACGCGATAAGTAATGTGAATTGCAGA<br>CACGTGAATCATCGAATCTTTGAACGCACCTTGCGCTCCT<br>TGGTGATCTGAGGAGCATGCCTGTTTGAGTGTCATTAAAT<br>TCTCAACCTAACCAACTTTTGGATAGGCTTGGATTGTGGG<br>GGCTTTTGCTGGCTTCAACCCCTCAGAAGTCAGCTCCCCT<br>TAAACGCATTAGTG
Agrocybe tuberosa	KF006385	AACTTTCAGCAACGGATCTCTTGGCTCTCGCATCGATGAA<br>GAACGCAGCGAAATGCGATAAGTAATGTGAATTGCAGAA<br>TTCAGTGAATCATCGAATCTTTGAACGCACCTTGCGCTCC<br>TTGGTATTCCGAGGAGCATGCCTGTTTGAGTGTCATTAAA<br>TTCTCAACCTTACCAGCTTGCTGATAATGGCTTGGATGTG<br>GGGGTCTTTTTGCTGGCTTCGGTCAGCTCCCCTTAAATGT<br>ATTAGCC

Notes: Data provided by the National Center for Biotechnology Information (NCBI).

Table S4. Number of incidents, patients, and deaths in mushroom poisoning outbreaks reported in Mainland China from 2010 to 2023.

Year	Number of incidents	Number of patients	Number of deaths	Mortality (%)
2010-2020	10036	38676	788	2.04
2021	327	923	20	2.17
2022	482	1332	28	2.10
2023	505	1303	16	1.23

Notes: Data provided by the Chinese Center for Disease Control and Prevention (CCDC).

Table S5. Number of incidents in various provinces, autonomous regions, and municipalities in Mainland China from 2010 to 2023.

First-level local state administrative organ	Total number of incidents
Yunnan Province	4281
Hunan Province	1952
Guizhou Province	1443
Sichuan Province	774
Jiangxi Province	364
Fujian Province	280
Guangxi Zhuang Autonomous Region	276
Zhejiang Province	238
Hubei Province	234
Shandong Province	212
Guangdong Province	208
Anhui Province	173
Chongqing Municipality	156
Jilin Province	114
Gansu Province	86
Ningxia Hui Autonomous Region	76
Hainan Province	64
Shanxi Province	63
Shaanxi Province	62
Jiangsu Province	58
Hebei Province	55
Henan Province	43
Inner Mongolia Autonomous Region	39
Xinjiang Uygur Autonomous Region	31
Heilongjiang Province	22
Liaoning Province	21
Beijing Municipality	14
Qinghai Province	4

Shanghai Municipality	3
Xizang Tibetan Autonomous Region	2
Tianjin Municipality	2

Notes: Data provided by CCDC.
